# Quantitative MRI comparison of multifidus muscle degeneration in thoracolumbar fractures treated with open and minimally invasive approach

**DOI:** 10.1186/s12891-018-2001-2

**Published:** 2018-03-07

**Authors:** F. Gilbert, T. M. Heintel, M. G. Jakubietz, H. Köstler, C. Sebald, R. H. Meffert, A. M. Weng

**Affiliations:** 1 Department of Trauma, Hand, Plastic and Reconstructive Surgery, Julius-Maximilians-University of Würzburg, Oberdürrbacherstr. 6, D-97080 Würzburg, Germany; 2 Department of Radiology Julius-Maximilians-University of Würzburg, Oberdürrbacherstr. 6, D-97080 Würzburg, Germany

**Keywords:** Spine trauma, Muscle degeneration, Minimal invasive surgery, Dorsal instrumentation

## Abstract

**Background:**

Minimally invasive pedicle screw fixation has less approach-related morbidity than open screw placement and is allegedly less traumatizing on paravertebral muscles, as there is no requirement to mobilize and retract the adjacent muscle portion. The approach-related long-term effects to the morphology of the paravertebral muscles are unknown. The purpose of this study was to compare the long-term amount of fatty degeneration of the multifidus muscle in patients treated with a classical open or a minimally invasive approach.

**Methods:**

Fourteen Patients meeting inclusion criteria were selected. In all patients a singular fracture of the thoracolumbar spine with a two-level posterior instrumentation was treated, either using an open approach or a minimally invasive approach. All patients underwent quantitative MRI spectroscopy for quantification of the fatty degeneration in the multifidus muscle as a long-term proof for muscle loss after minimum 4-year follow-up. Clinical outcome was assessed using Oswestry Low Back Pain Disability Questionnaire, SF-36 and VA-scale for pain.

**Results:**

The minimally invasive approach group failed to show less muscle degeneration in comparison to the open group. Total amount of fatty degeneration was 14.22% in the MIS group and 12.60% in the open group (*p* = 0.64). In accordance to MRI quantitative results there was no difference in the clinical outcome after a mean follow up of 5.9 years (±1.8).

**Conclusion:**

As short-term advantages of minimal invasive screw placement have been widely demonstrated, no advantage of the MIS, displaying a significant difference in the amount of fatty degeneration and resulting in a better clinical outcome could be found. Besides the well-known short-term advantage of minimally invasive pedicle screw placement, a long-term advantage, such as less muscle degeneration and thus superior clinical results, compared to the open approach could not be shown.

## Background

Minimally invasive pedicle screw placement is an equally safe and reproducible procedure for treatment of thoracolumbar fractures in comparison to the open approach [9] .Advantages of a minimally invasive approach are less blood loss, lower muscle trauma and a lower rate of surgical site infection than in open approach [2, 6, 18].

Muscle trauma in open approach results from sharp, subperiosteal dissection of the multifidi and rotator muscles, blunt force trough retraction and damage of the posterior branches of the spinal nerves leading to loss of function of the motor unit. The nerve damage has been shown to be solely approach related and unlikely to be caused by the trauma itself [6, 12]. EMG studies have shown that pathological changes in the muscle innervation after open approach are persistent in midterm follow up and that reduction of the surface EMG signal correlates negatively to the amount of pain. EMG analysis has shown that an open approach leads to pathological innervation patterns of the paravertebral muscles in the long-term [6, 12]. While EMG studies may be a strong predictor of muscle degeneration, the actual true amount of degeneration, visible as fatty degeneration, has not been evaluated quantitatively. Chronic denervation leads to MRI detectable muscle degeneration and fatty infiltration. Whether the amount of fatty degeneration of the paravertebral muscles is further influenced by segmental immobilization is unclear, thus patients, permanently immobilized by implants must not be included in such studies [13]. Apart from an effect in the EMG studies, it remains unclear whether a higher amount of muscle damage in the open approach has long-term effects on the morphology of paravertebral spine muscles, and if there is an approach depended difference in morphology of the paravertebral muscles. Fatty degeneration of the paravertebral muscles has been described even in asymptomatic individuals, thus after surgery an increased degeneration would be expected. The goal of the study was a quantitative evaluation of fatty degeneration in the paravertebral muscles in either an open or a minimal invasive approach in the long term.

## Methods

A statistical power analysis was performed using GLMPOWER in SAS 9.3 (SAS Institute Inc., North Carolina, USA), furthermore a 2-way-covariant analysis with ANCOVA for a level of significance of 0.05 was performed. A total number of 6 patients for each group were calculated to detect significant differences related to the type of surgery with a power of 90%. The expectable variation in both groups were based on the findings of Fan et al. with a comparable study design for the investigation of mono-segmental dorsal instrumentation in degenerative spine diseases. The institutional review board approved the study (Etic committee of the Julius-Maximilians-University, Würzburg, Germany approval number 55/15) and written informed consent was obtained from each patient before participation.

The spine register of level 1 Trauma center between 2007 and 2012 included 963 patients who underwent spine surgery for fractures of the thoracolumbar spine. The exclusion criteria were, among others, remaining osteosynthesis material (interference with MRI fat measurement), concomitant neuro trauma (Frankel A-D), osteoporotic fractures, low energy trauma, muscle diseases, Diabetes, use of steroids, obesity (BMI > 30) and claustrophobia.

After application of the inclusion criteria, monosegemental fractures of the thoracolumbar spine from T 11 to L2, treated with a bisegmental dorsal instrumentation, minimal follow up time of 48 months, age 18–65 and Frankel E classification, 34 patients that met inclusion were identified for the retrospective analyzation. Furthermore, two patients had died due to unrelated causes, 6 had moved away and 12 refused participation due to claustrophobia or had contraindications for MRI (pacer, defibrillator). 14 Patients were available for the investigation.

The level of pain was assessed using VAS spine score. For further evaluation of lower back pain the Oswestry Low Back Pain Disability Questionnaire and the SF-36 were surveyed. The fractures were classified according to the AO/Magerl classification [15]. Estimated blood loss and operation time were retrospectively obtained from the anesthesiology protocol of the clinical databank.

A postoperative CT scan was available for all patients, accuracy of pedicles screw placement was analyzed using a pedicle screw scoring system and sagittal alignment was measured [22].

**Fig. 1:**
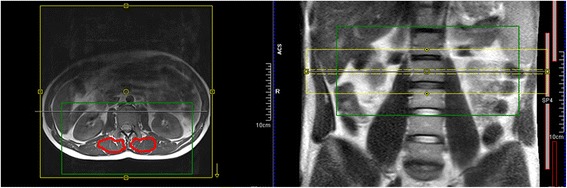
A quantitative MR-spectroscopy was performed and the fat amount in the multifidus muscle was measured at the fracture level and the adjacent vetebrae

In every patient a MRI with a spectroscopy using the SPLASH technique (spectroscopic fast low angle shot) was performed, which allows an exact quantification of fat in a 2-D MRI layer. The exact technique and the reliability of the spectroscopy was described by our workgroup previously [5, 11]. Borders of the multifidus muscle were delineated manually as custom-made software allows measuring in a random shaped region of interest. The amount of fatty degeneration was measured at the fracture level and in the middle of the adjacent vertebras (Fig. 1). The mean values and standard deviation of the FD were calculated.

Statistical analyses were performed using Excel and SPSS. Level of significance was calculated using the independent samples Mann-Whitney U-test and the Kruskal-Wallis test.

## Results

In the open group all patients were treated using a Schanz-screw system (USS fracture Depuy-Synthes) MIS group all patients were treated with a toploader system with poly-axial screws (Longitude Horizon Fa Medtronic). Patients in the open group had a mean age of 46.5 years (±16.3) and in the MIS group an age of 41.3 (±17.6) years (*p* = 0.616). Blood loss was significantly lower in the MIS group and operation time did not show any differences between the two groups. The other baseline characteristics like BMI, follow up time, sex and smoker status did not show any significant difference (table 1). Fracture distribution for the open group revealed five A3 fractures, two B1 and two B3 fractures. In the MIS group four A3 and two B2 fractures were observed. Accuracy of pedicel screw placement revealed a good or excellent position for all screws according to the pedicle screw rating system of Zdichavsky et al. [21].

**Table 1:** Patients demographic data and results

	All *n* = 14	Open *n* = 8	MIS *n* = 6	*p*-value
Age [years]	44.3 (±17.1)	46.5 (16.3)	41.3 (±17.6)	0.616
Sex m:f	8:6	5:3	3:3	0.67
BMI	26.9 (±2.3)	27.1 (±2.1)	26.7 (±2.6)	0.75
Smoker	10	5	5	
Follow up Time [years]	5.9 (±1.8)	6.2 (±2.1)	5.43 (±1.1)	0.438
Amount of FD[%]	13.3 (±6.0)	12.6 (±6.8)	14.22 (±5.9)	0.642
Oswestry [%]	8 (±1)	6 (±1)	12 (±1)	0.134
SF 36	84 (±10.7)	86.3 (±8.7)	81 (±12.5)	0.51
VAS	1 (±1.2)	0.95 (±1.3)	1 (±1.1)	0.9
Blood loss [ml]	254 (±46)	110 (±23)	350 (±96)	0.0007
Operation Time [min]	98 (±14)	102 (±17)	97 (±12)	0.82

Mean loss of sagittal profile was 7° (±1.7) in the open and 6.8°(±1.7) in the MIS group compared from the initial postoperative lateral x-ray compared to the latest available x ray (*p* = 0.73). Implant removal was performed after a mean of 1.5 (±1.2) years in group A and after 1.3 (±1.4) years in group B (*p* = 0.86).

Functional assessment did not show a significant difference between the two groups. Results of the functional scores are shown in table 2. The result for the Oswestry Low Back Pain Disability Questionnaire for all individuals was 9% which resembles an excellent result. For the open group this score was 6% and for MIS group it was 12% without showing a significant difference *p* = 0.13. For the SF 36 and the VAS spine score no significant differences were detected between the two groups.

**Table 2:** Inclusion and exclusion criteria for the study

Inclusion criteria	
Monosegemental fractures of the thoracolumbar spine from T 11 to L2 which were treated with a bisegmental dorsal instrumentation Minimal follow up time 48 months Age 18–65 Frankel E	
Exclusion criteria	
Metal implants in the fracture region Neurological deficits (Frankel A-D) Osteoporotic fractures, low energy trauma Muscle diseases Diabetes, Steroid, Adipositas >BMI 30 Claustrophobia Contraindications for MRI	

Finger floor distance was 5 cm (±4.7) in the MIS and 5.25 cm (±2.4) in the open group.

**Fig. 2:**
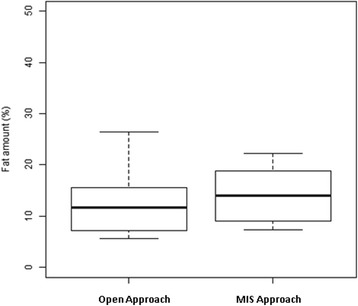
MR-spectroscopy revealed a fat amount of 12.6% (±6.3) for the open and a amount 14.2% (±5.3) for the MIS group. The upper and the lower whisker of boxplots represent the standard deviation. The heights of box represents the interquartile range (25% and 75% quartile). The line in the middle represents the median

MR-spectroscopy of the paravertebral muscles revealed a mean fat amount of 13.3% (±6.1%) for all individuals. Patients treated with an open approach (*n* = 8) had a mean fat amount of 12.6% (±6.4%) and Patients with a minimal-invasive approach (*n* = 6) had a mean fat amount of 14.2% (±5.4%) (Fig. 2). Difference did not reach the level of significance (*p* = 0.642).

No implant related complications were observed in the two cohorts.

## Discussion

### Advantages of MIS

**Fig. 3:**
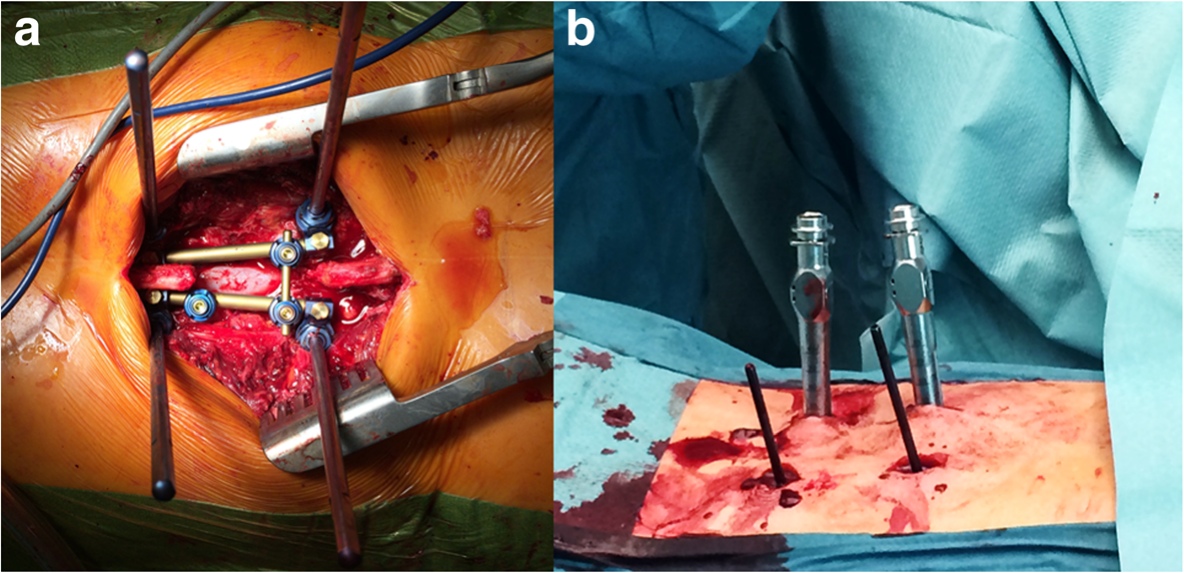
Open approach (**a**) for pedicle screw placement and Minimal invasive approach (**b**)

The advantages of minimally-invasive approaches in spine surgery are well investigated and accepted. As a muscle sparing technique, it reduces the approach-related morbidity. In particular use of minimal invasive approaches has less blood loss, lower infection rates, less operation time, faster recovery and a decreased serum concentration of creatinkinase, by providing the same safety and accuracy as open techniques [8, 9]. These effects on muscle have been supported by EMG studies showing a significant reduction of pathological EMG patterns if a minimal invasive approach was used in mid-term investigations [6]. A reduction of muscular disconnection from the bone, a reduction of nerve damage and the reduction of blunt trauma trough retractors are assumed to be beneficial (Fig. 3) [6, 18]. Long term effects of open dorsal approaches have been described, as Kramer et al. found pathological EMG patterns in the muscles of the whole erector spinae after a follow up time of 4 years in a cohort of 32 patients who underwent open dorsal pedicle screw placement for fracture stabilization [12]. Patients with minimal invasive approaches were not investigated in this study; so, it remains unclear if these changes persist over a longer period and whether EMG changes translate into quantifiable effects on muscle diameter and ultimately atrophy.

Despite its disadvantages open approaches are still widely used and necessary especially in trauma with concomitant neurological deficit for decompression or if direct reduction of intra-spinal fragments is desired.

### Fatty degeneration of paravertebral muscles and influencing factors

**Fig. 4:**
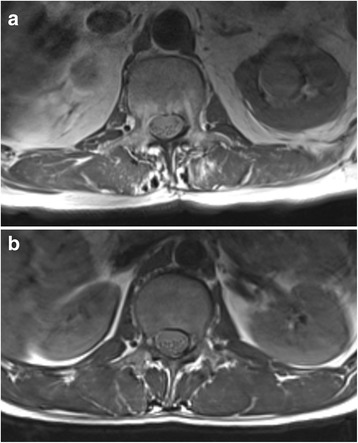
T1-weighted transversal imagfe of the paravertebral muscles. MR spectroscopy reveald a fat amount of 26.5% for the multifidus muscle in **a** and an amount of 5.6% in **b**

As muscular atrophy and degeneration is a promotor of lower back pain, which has been shown trough biopsy and MRI analysis of the lumbar muscles, it is unknown if there is an approach-related impact to FD of the paravertebral muscles in a trauma context [10, 20]. Various quantitative and semi-quantitative MRI methods are employed for analysis of amount of FD, but there is no standardized protocol yet, thus comparison of the different studies is limited. As the relation between the amount of FD and the occurrence of low back pain is well documented, several influencing factors for promoting FD have been described. In detail age, sex, spinal degeneration i.e. lumbar disc herniation, spondylolisthesis and facet joint arthritis influence the amount of FD (Fig. 4). [3, 4, 14, 19]. This is supported by histological findings in chronical lumbar spine diseases showing an increase of degenerative changes in the multifidus muscles which occur as decrease of the cross sectional area, reduction of type II fibers, signs of inflammation and decreased vascularity [20]. Thus, an increased amount of fatty degeneration should be expected in patients with fractures that are furthermore treated with an open method in contrast to minimally invasive surgery. Surprisingly the long-term morphological changes in the paravertebral muscles were not approach related in this study and where approximately in the range of healthy individuals found in other spectroscopic MRI and semiquantitative studies [1, 7, 16]. MRI and biopsy studies showed that FD of the paravertebral muscles occurs regularly after spine surgery or with degenerative changes of the spine, even if segmental immobilization is performed trough anterior techniques alone. This suggests that the changes in morphology seem to be mainly triggered by the segmental immobilization, age and degenerative changes than the nerve damage through surgery [1, 3, 17]. Studies using MRI quantification to assess the lumbar spine muscles are rare in trauma situation. In contradiction to our findings Fan et al. found a significant reduction of in the cross-sectional area of the multifidus muscle postoperatively when an open approach was used for dorsal mono-segmental fusion (PLIF) in a cohort of 21 patients after 1 year. The follow up time of 1 year was shorter, the patient cohort was about 10 years older, the focus was on surgery for degenerative changes and a semi-quantitative method for analyzing the amount of FD was used [2]. Therefore, comparison may not be possible. As reinnveration potential has been demonstrated in EMG studies, the effect of the approach may be attenuated in the long term and could lead to a remodeling of the muscle structure [3, 6].

The relatively small number of participants and the retrospective design of the study do not allow conclusions about other influencing factors on the amount of FD. A prospective design would be helpful how FD develops over time, and if FD is a reversible process in the paravertebral muscles.

## Conclusion

In this retrospective study setting, we did not find any differences in approach related long-term effects to the morphology of the paravertebral muscles. Studies focusing on the morphology of the paravertebral muscles in a trauma setting are rare, thus this study may serve as an approach to quantitative assessment of muscle morphology after spine trauma surgery. With a prospective design the impact of the surgical approach to the muscle and development of FD over time may be better understood. Nevertheless, we did not find approach-related the long-term effects on muscle morphology in this cohort of trauma patients.

## Abbreviations

BMI: Body mass index
EMG: Electromyography
FD: Fatty Degeneration
MIS: Minimally invasive surgery
mm: millimeter
MRI: Magnet Resonance Imaging
SPLASH: Spectroscopic Fast Low Angle Shot
PACS: Picture archiving and communication system
VAS: Visual analog scale
